# Serum vs. Dietary Magnesium and Cardiometabolic Outcomes in Saudi Adolescents

**DOI:** 10.3390/nu18193302

**Published:** 2026-10-08

**Authors:** Sobhy M. Yakout, Malak Nawaz K. Khattak, Abdullah M. Alnaami, Shaun Sabico, Nicola Veronese, Mario Barbagallo, Nasser M. Al-Daghri

**Affiliations:** 1 Biochemistry Department, College of Science, King Saud University, Riyadh 11451, Saudi Arabia; 2 Geriatric Unit, Department of Internal Medicine and Geriatrics, Faculty of Medicine, Saint Camillus International University of Health Sciences, 00131 Rome, Italy; 3 Geriatric Unit, Department of Internal Medicine and Geriatrics, University of Palermo, 90100 Palermo, Italy

**Keywords:** magnesium, hypomagnesemia, adolescents, cardiometabolic health, obesity, dietary intake, Saudi Arabia

## Abstract

**Background/Objectives**: Magnesium (Mg) is an essential micronutrient that participates in more than 300 enzymatic reactions, with well-established functions in glucose and lipid metabolism and cardiovascular health. Although serum Mg is routinely used in clinical practice, circulating concentrations may not accurately represent total body Mg status because of tight physiological regulation. The present study evaluated the associations of serum Mg concentration and dietary Mg intake with cardiometabolic outcomes in a cohort of Saudi adolescents. **Methods**: This cross-sectional study involved 234 Saudi adolescents (166 girls, 68 boys; mean age 14.8 ± 1.7 years) who were assessed for anthropometrics, biochemical parameters and dietary intake. Hypomagnesemia was classified as serum Mg <0.75 mmol/L, while inadequate dietary Mg intake was based on age- and sex-specific cutoffs for children. **Results**: Hypomagnesemia was present in 87 participants (37.2%) and was more common among boys than girls (50% vs. 31.9%, *p* = 0.011). No significant differences in the assessed cardiometabolic measurements were observed across serum Mg tertiles. Absolute dietary Mg intake was strongly correlated with total energy and other dietary intakes and was inversely associated with body mass index. Associations based on absolute dietary Mg intake were attenuated after energy adjustment for overweight/obesity. For elevated triglycerides, the highest vs. lowest tertile of residual-adjusted Mg showed a nominal inverse association in the age- and sex-adjusted model (OR: 0.40; 95% CI: 0.17–0.96). However, the corresponding trend did not remain statistically significant after false discovery rate correction. **Conclusions**: Serum Mg showed limited associations with cardiometabolic outcomes, while associations between dietary Mg and cardiometabolic outcomes were sensitive to energy adjustment and multiple-comparison correction. The inverse association between residual-adjusted Mg and elevated triglycerides should therefore be considered exploratory. These findings highlight potential differences between serum and dietary Mg as indicators of Mg status and underscore the importance of accounting for total energy intake when examining dietary Mg in relation to cardiometabolic health in adolescents.

## 1. Introduction

Cardiometabolic diseases, which include obesity, type 2 diabetes mellitus, dyslipidemia, and hypertension, have emerged as defining public health challenges of the 21st century, with alarming upward trends now extending into childhood and adolescence [1]. The burden is particularly acute in the Arabian Peninsula, where Saudi Arabia reports adolescent obesity prevalence ranging from 20% to 35%, driven by rapid nutritional transition towards energy-dense, micronutrient-poor dietary patterns, combined with increasingly sedentary lifestyles [2,3]. Cardiometabolic abnormalities established during adolescence are known to persist into adulthood, substantially compounding lifetime cardiovascular disease risk [4]. Identifying modifiable nutritional determinants of these outcomes during the adolescent phase therefore carries considerable preventive potential.

Magnesium (Mg) is the fourth most abundant cation in the human body and the second most abundant intracellular cation, functioning as an obligatory cofactor for more than 300 enzymatic reactions involved in energy metabolism, protein synthesis, nucleic acid stability, and transmembrane ion transport [5,6]. Furthermore, Mg plays a pivotal role in insulin signal transduction: it is required for the autophosphorylation of the insulin receptor tyrosine kinase, facilitates glucose transporter (GLUT-4) translocation to the cell membrane, and modulates pancreatic β-cell secretory function [7,8]. Adequate Mg availability contributes to vascular function by limiting calcium (Ca)-mediated contraction of vascular smooth muscle, thereby facilitating vasodilation and maintaining blood pressure homeostasis [9,10]. Ca and Mg also have closely coordinated roles in cardiac function, particularly in cardiomyocyte excitation–contraction coupling and the maintenance of normal cardiac electrical activity, highlighting the importance of their physiological balance for cardiovascular homeostasis [11]. Mg is likewise involved in lipid metabolism through its role in the activity of lecithin–cholesterol acyltransferase (LCAT) and lipoprotein lipase, enzymes that regulate HDL maturation and triglyceride catabolism [12]. Furthermore, Mg suppresses nuclear factor-κB (NF-κB) activation and reduces pro-inflammatory cytokine production (IL-6, TNF-α), linking Mg deficiency to chronic low-grade inflammation that triggers insulin resistance and atherogenesis [13,14].

Mg homeostasis is maintained through a tightly regulated interplay of intestinal absorption (primarily in the small intestine via TRPM6/TRPM7 channels), renal reabsorption (predominantly in the thick ascending limb of the loop of Henle and the distal convoluted tubule), and skeletal buffering, with approximately 60% of total body Mg stored in bone [5]. Serum Mg reflects approximately only 0.3% of total body Mg. Owing to tight physiological regulation, circulating concentrations usually remain within the normal range (0.75–0.95 mmol/L), even when intracellular or tissue Mg stores have declined substantially. Consequently, serum Mg is widely regarded as an insensitive marker of true Mg status, and a “functional” or “latent” Mg deficiency may exist even in apparently normal serum values [5,6]. This dissociation between circulating and tissue Mg levels complicates clinical interpretation of serum-based measurements and may partly explain the inconsistencies observed across epidemiological studies.

Rapid growth and developmental changes during adolescence are accompanied by increased Mg requirements, making this stage of life especially vulnerable to inadequate Mg intake. Rapid skeletal growth during puberty substantially increases Mg requirements (estimated adequate intake: 360–410 mg/day for ages 14–18 years), while concurrent dietary shifts towards processed, low-Mg foods reduce dietary supply [6]. Hormonal changes, particularly rising insulin-like growth factor-1 (IGF-1) and sex steroids, alter renal Mg handling, and the widespread vitamin D insufficiency, which is prevalent among Saudi adolescents, may also influence Mg homeostasis and intestinal Mg handling [5]. Together, these factors may predispose adolescents to subclinical Mg depletion, with potential implications for cardiometabolic health.

Both low Mg status and inadequate intake have been associated with cardiometabolic abnormalities [14,15,16,17]. However, the literature on the associations of circulating and dietary Mg with cardiometabolic outcomes in adolescents remains limited and methodologically heterogeneous. Many published studies have focused primarily on serum Mg, with dietary Mg intake less frequently evaluated as a separate exposure. Moreover, few studies have concurrently assessed serum and dietary Mg within the same adolescent cohort, limiting direct comparison of their associations with cardiometabolic outcomes. Among adult populations, inverse associations between dietary Mg and metabolic syndrome (MetS) components have been reported [14,15,16,17], yet data in adolescent populations, particularly from the Middle Eastern region, are sparse [18,19].

Saudi adolescents represent a particularly informative study population to fill this gap. They combine high rates of cardiometabolic outcomes, widespread micronutrient insufficiency (including Mg, vitamin D, and iron), a dietary pattern increasingly characterized by high intakes of refined carbohydrates and saturated fats with low fruit, vegetable, and whole-grain consumption, and a genetic background that may confer differential susceptibility to insulin resistance and dyslipidemia [2,3]. Identifying the associations between nutritional factors and cardiometabolic health in this population may improve our understanding of adolescent cardiometabolic health.

Therefore, this study aimed to: (1) determine the prevalence of hypomagnesemia and inadequate dietary Mg intake in Saudi adolescents; (2) examine the relationships between serum and dietary Mg with cardiometabolic and dietary parameters; and (3) assess their associations with cardiometabolic outcomes, including analyses accounting for total energy intake. By evaluating both markers within the same cohort, the study sought to clarify their potential distinct associations with cardiometabolic health in Arab adolescents.

## 2. Materials and Methods

### 2.1. Study Design and Participants

This cross-sectional study was conducted among adolescents aged 12–18 years recruited from secondary schools in Saudi Arabia as part of a wider Riyadh School Health Survey [20,21]. Eligible participants were recruited consecutively throughout one academic year. Exclusion criteria included: known endocrine disorders (diabetes mellitus, thyroid disease, or growth hormone deficiency), chronic kidney or gastrointestinal disease, use of medications known to affect Mg metabolism (diuretics, proton-pump inhibitors, aminoglycosides), pregnancy, and incomplete data. Analyses were based on 234 adolescents (166 girls and 68 boys). The study protocol was approved by the Institutional Review Board (IRB) of the College of Medicine, King Saud University (E-25-9801, approved 18 June 2025), and all procedures were conducted in accordance with the Declaration of Helsinki. Assents were obtained from all adolescent participants, and written informed consent was obtained from their parents or legal guardians prior to enrolment. Participants also completed a comprehensive questionnaire that included demographic information and medical history.

### 2.2. Anthropometric Measurements

Body weight was measured to the nearest 0.1 kg using a calibrated digital scale (SECA 813, Hamburg, Germany) with participants wearing light clothing and no shoes. Standing height was measured to the nearest 0.1 cm using a wall-mounted stadiometer. Body mass index (BMI) was calculated as weight (kg)/height (m)^2^. Waist circumference was measured to the nearest 0.1 cm at the midpoint between the lower costal margin and the iliac crest at end-expiration. Hip circumference was measured to the nearest 0.1 cm at the widest portion of the buttocks. Waist-to-hip ratio (WHR) was calculated as waist circumference divided by hip circumference. Two seated blood pressure readings were obtained after a 5-min rest using an automated oscillometric device (Omron Healthcare, Kyoto, Japan). The average of the two readings was used for analysis.

### 2.3. Biochemical Analysis

Following an overnight fast of at least 8 h, venous blood samples were obtained for biochemical analyses. Fasting blood glucose (FBG), total cholesterol (TC), high-density lipoprotein cholesterol (HDL-C), and triglycerides (TG) were determined by enzymatic methods using a Konelab 20XT clinical chemistry analyzer (Thermo Scientific, Vantaa, Finland) and commercially available assay kits. HbA1c was assessed by ion-exchange high-performance liquid chromatography on a D-10 Hemoglobin Analyzer (Bio-Rad Laboratories, Hercules, CA, USA). Serum Mg was measured using a Konelab 20XT clinical chemistry analyzer (Thermo Scientific, Vantaa, Finland) using a colorimetric Xylidyl Blue I method with Thermo Scientific Magnesium reagent (kit code 981884/981885). In this assay, Mg forms a red complex with Xylidyl Blue I in an alkaline medium, and the absorbance measured at 510 nm is proportional to the Mg concentration; Ca interference is minimized by ethylene glycol tetraacetic acid (EGTA). Calibration was performed using sCal (code 981831), according to the manufacturer’s instructions. Internal quality control was conducted using Nortrol (code 981043) and Abtrol (code 981044), while external quality control was performed in accordance with the laboratory’s routine quality assurance procedures. The intra-assay and inter-assay coefficients of variation were 1.1–1.5% and 2.9–3.7%, respectively. The analytical limit of detection (LoD) was 0.03 mmol/L, and the limit of quantification (LoQ) was 0.05 mmol/L. Three serum Mg measurements were below the LoD, and only 4 measurements were below the LoQ. The 4 Mg measurements below LoQ were retained in the primary analyses (n = 234), without substitution. Supplementary Table S1 presents a descriptive comparison of serum Mg distributions with and without these measurements (n = 230 after exclusion; Figure S1). This descriptive comparison does not assess the influence of these measurements on the inferential analyses. Values below the LoQ have greater analytical uncertainty, which should be considered when interpreting the findings. A serum Mg concentration <0.75 mmol/L was used to define hypomagnesemia, a threshold previously applied in a population-based study of adolescents aged 12–19 years [22]. All sample analyses were carried out at the Chair for Biomarkers of Chronic Diseases (CBCD) in King Saud University, Riyadh, Saudi Arabia.

### 2.4. Dietary Assessment

Dietary intake was assessed using a validated, culturally adapted semi-quantitative food frequency questionnaire (FFQ) designed for the Saudi population [23]. The FFQ captured habitual intake over the preceding three months across 150 food items with nine frequency categories (from never to ≥6 times/day). Portion sizes were estimated using standard household measures and food photographs. Nutrient composition was calculated using the Saudi Food Composition Database supplemented by the USDA National Nutrient Database. For the present analysis, inadequate dietary Mg intake was defined as estimated average requirement (EAR) ≤200 mg/day for boys and girls aged 11–13; ≤340 mg/day for boys aged 14–18 and <300 mg/day for girls aged 14–18 [6,24]. To account for variations in total energy intake (distribution in Supplementary Table S2), dietary Mg was additionally evaluated using two energy-adjustment approaches. Mg density was calculated as dietary Mg intake (mg/day) divided by total energy intake (kcal/day), multiplied by 1000 and expressed as mg/1000 kcal. Energy-adjusted Mg intake was also estimated using the residual method, in which dietary Mg intake was regressed on total energy intake and the resulting residuals, representing the variation in Mg intake independent of total energy intake, were used in subsequent analyses.

### 2.5. Definition of Cardiometabolic Outcomes

Cardiometabolic outcomes for the primary analysis were defined using Cook’s definition [25,26] as follows: (1) overweight/obesity: BMI ≥ 85th percentile for age and sex according to the CDC growth charts [27]; (2) central obesity: WC > 90th percentile for age and sex (3) elevated blood pressure: >90th percentile for age, and sex (4) elevated glucose: FBG ≥ 6.1 mmol/L; (5) low HDL-C: <1.03 mmol/L; and (6) elevated triglycerides: TG of ≥1.24 mmol/L for ages 10–15 years; TG ≥ 1.7 mmol/L for ≥16 years. For the sensitivity analysis, cardiometabolic outcomes were additionally defined according to the International Diabetes Federation (IDF) pediatric/adolescent criteria, and the associations of energy-adjusted dietary Mg, assessed using both the density and residual methods, with these outcomes were re-evaluated using logistic regression [28]. The IDF cut-offs were central obesity: WC ≥90th percentile, age- and sex-specific (10 to <16 years), adult waist criterion at ≥16 years (WC ≥ 94 cm for Europid males and ≥80 cm for Europid females, with ethnic-specific values for other groups); elevated blood pressure: SBP ≥ 130 mmHg or DBP ≥ 85 mmHg; elevated glucose: FBG ≥ 5.6 mmol/L; low HDL-C: HDL-C < 1.03 mmol/L for boys and girls 10 to <16 years old; HDL-C < 1.03 mmol/L for boys and <1.29 mmol/L for girls for 16 years and above; and elevated triglycerides: TG ≥ 1.7 mmol/L.

### 2.6. Statistical Analysis

Statistical analyses were performed using SPSS version 26.0 (IBM Corp., Armonk, NY, USA). Data distribution was assessed using the Kolmogorov–Smirnov test. Continuous variables are presented as mean ± standard deviation (SD) for normally distributed data or median (interquartile range [IQR]) for non-normally distributed data, whereas categorical variables are expressed as frequencies and percentages. Comparisons between two groups were performed using the independent-samples *t* test or the Mann–Whitney U test, as appropriate, while categorical variables were compared using the chi-square test. To account for total energy intake, dietary Mg was additionally analyzed using the Mg-density and energy-adjusted residual measures described above. Spearman rank correlation analysis was used to examine the associations of serum Mg concentration and dietary Mg intake with clinical, biochemical, and dietary variables. Participants were categorized into tertiles of serum Mg concentration and dietary Mg intake. For energy-adjusted analyses, Mg-density and residual-adjusted Mg measures were also categorized into tertiles. Differences across tertiles were assessed using one-way analysis of variance (ANOVA) for normally distributed variables or the Kruskal–Wallis test for non-normally distributed variables. Multivariable logistic regression models were used to estimate odds ratios (ORs) and 95% confidence intervals (CIs) for cardiometabolic outcomes, using the lowest tertile (T1) as the reference category. Adjusted models included age and sex. Additional sensitivity models were further adjusted for total energy intake (kcal/day). Tests for linear trend across tertiles were performed by entering the tertile as an ordinal variable in the logistic regression models.

Implausible energy reporters were identified based on the ratio of reported energy intake to estimated basal metabolic rate (EI:BMR), using predefined Goldberg-type cut-offs. Participants with EI:BMR < 1.35 or ≥2.40 were classified as implausible energy reporters. The BMR equations and detailed classification criteria are provided in Supplementary Methods S1. To account for multiple comparisons, the Benjamini–Hochberg false discovery rate (FDR) procedure was applied to the *p*-values from the trend analyses. FDR-adjusted q-values are reported alongside the corresponding nominal *p*-values. FDR correction was applied separately across the six cardiometabolic outcome trend tests for each Mg measure within each model specification. Statistical tests were two-tailed. A *p* < 0.05 was considered statistically significant for individual analyses, while FDR-adjusted q < 0.05 was considered statistically significant after correction for multiple comparisons.

## 3. Results

### 3.1. Participant Characteristics and Serum Mg Status

The study included 234 Saudi adolescents with a mean age of 14.8 ± 1.7 years [166 girls (70.9%) and 68 boys (29.1%)]. Mean BMI was 22.9 ± 5.3 kg/m^2^, with 39.0% of the cohort classified as overweight or obese. Table 1 presents the clinical and dietary characteristics stratified according to sex and serum Mg status. Across the sex-stratified analyses, age, BMI, waist-to-hip ratio (WHR), and blood pressure were comparable between adolescents with normomagnesemia and hypomagnesemia (all *p* > 0.05). Fasting blood glucose, HbA1c, total cholesterol, and triglycerides also did not differ significantly between serum groups. HDL-C was the only cardiometabolic parameter that differed significantly: adolescents with hypomagnesemia had lower HDL-C concentrations than those with normomagnesemia (1.01 ± 0.3 vs. 1.12 ± 0.4 mmol/L, *p* = 0.037), with the difference also observed among boys (1.05 ± 0.3 vs. 1.22 ± 0.4 mmol/L, *p* = 0.04).

Several dietary intake variables differed according to serum Mg status. In the overall sample, adolescents with hypomagnesemia reported lower median intakes of energy (*p* = 0.021), fat energy (*p* = 0.007), protein (*p* = 0.048), omega-3 fatty acids (*p* = 0.007), and omega-6 fatty acids (*p* = 0.003). Among boys, hypomagnesemia was also associated with lower reported intakes of energy (*p* = 0.007), fat energy (*p* = 0.004), protein (*p* = 0.011), carbohydrates (*p* = 0.043), omega-3 fatty acids (*p* = 0.002), and omega-6 fatty acids (*p* = 0.005). No significant differences in dietary intake variables were observed among girls according to serum Mg status (all *p* > 0.05).

### 3.2. Prevalence of Hypomagnesemia and Inadequate Dietary Mg Intake

**Figure 1:**
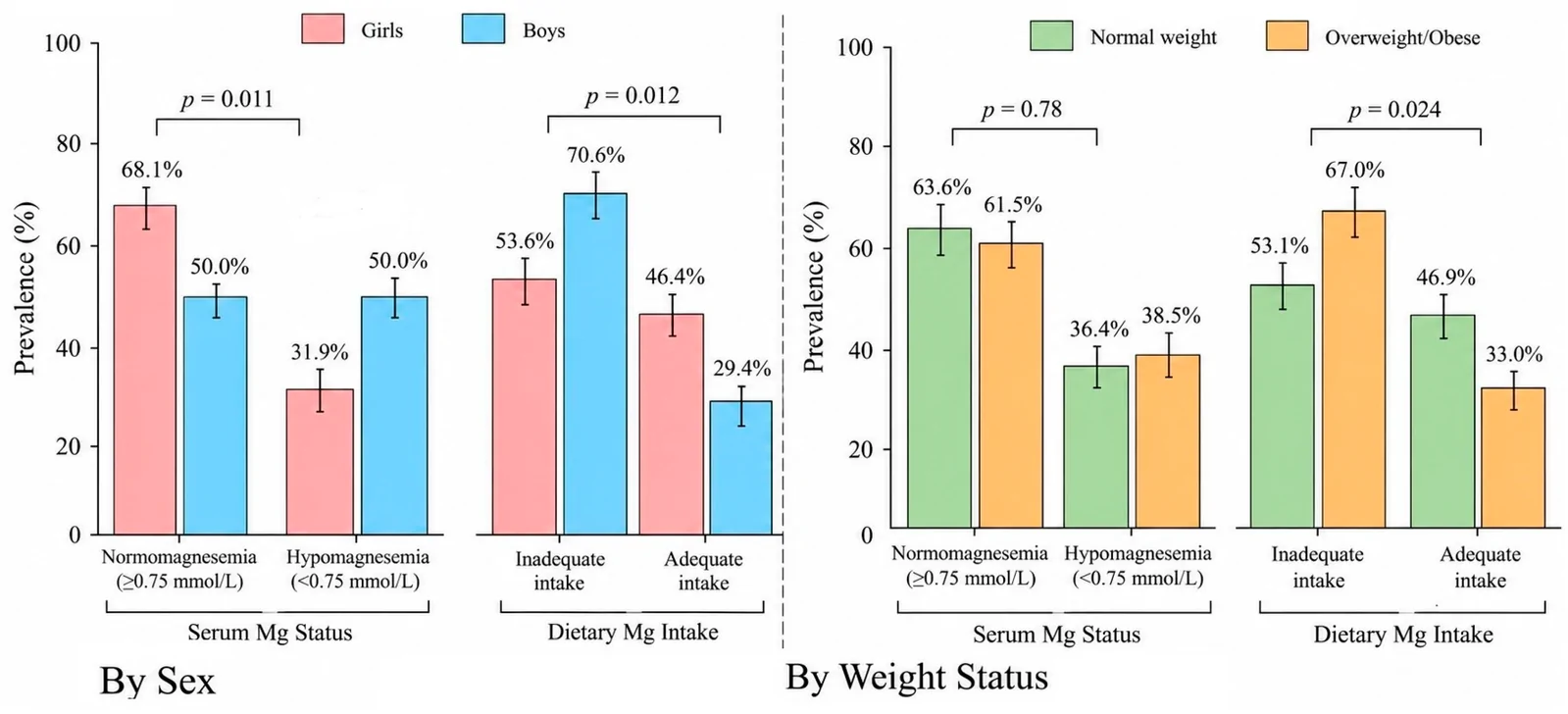
Prevalence of hypomagnesemia (serum Mg < 0.75 mmol/L) and inadequate dietary Mg intake stratified by sex (girls vs. boys) and weight status (normal weight vs. overweight/obese) among 234 Saudi adolescents. Data presented as percentages (%).

The prevalence of hypomagnesemia and inadequate dietary Mg intake, stratified by sex and weight status, is presented in Table 2 and illustrated in Figure 1. Hypomagnesemia was identified in 87 participants (37.2%) and was significantly more prevalent among boys than girls (50% vs. 31.9%, respectively; *p* = 0.011). No significant difference in the prevalence of hypomagnesemia was observed according to weight status (*p* = 0.78). Inadequate Mg intake was more prevalent among boys than girls (70.6% vs. 53.6%; *p* = 0.012) and among overweight than normal weight (67% vs. 53.1%; *p* = 0.024). Serum Mg concentration and dietary Mg intake were not correlated (r = 0.07, *p* > 0.05).

### 3.3. Correlation Analysis

Spearman correlation coefficients between serum Mg, dietary Mg, energy-adjusted Mg measures, and selected cardiometabolic and dietary parameters are presented in Table 3 and Table 4. Serum Mg showed weak and largely non-significant correlations with the assessed cardiometabolic and dietary parameters in the overall sample. Weak positive correlations were observed with omega-3 (r = 0.140, *p* < 0.05) and omega-6 fatty acid intake (r = 0.155, *p* < 0.05), while the correlation between serum and dietary Mg was weak and non-significant (r = 0.07, *p* > 0.05). Absolute dietary Mg intake was strongly positively correlated with total energy intake (r = 0.867), protein (r = 0.902), carbohydrates (r = 0.835), fat energy (r = 0.801), dietary fiber (r = 0.836), omega-3 fatty acids (r = 0.779), and omega-6 fatty acids (r = 0.744) (all *p* < 0.01). Dietary Mg intake was inversely correlated with age (r = −0.230, *p* < 0.01) and BMI (r = −0.158, *p* < 0.05). After accounting for energy intake, the correlations with BMI were attenuated and were no longer statistically significant for either Mg density (r = −0.06) or energy-adjusted Mg residuals (r = −0.11). Mg density and Mg residuals remained correlated with absolute dietary Mg intake (r = 0.33 and r = 0.61, respectively; both *p* < 0.01), and were strongly correlated with each other (r = 0.87, *p* < 0.01).

### 3.4. Clinical and Dietary Characteristics Across Mg Tertiles

Clinical and dietary characteristics across serum and dietary Mg tertiles are presented in Table 5. No significant differences in BMI, WHR, blood pressure, fasting blood glucose, HbA1c, total cholesterol, HDL-C, or triglycerides were observed across serum Mg tertiles (all *p* > 0.05). Among dietary variables, significant differences across serum Mg tertiles were observed for fat energy (*p* = 0.03), omega-3 fatty acid intake (*p* = 0.013), and omega-6 fatty acid intake (*p* = 0.007), whereas the difference in total energy intake did not reach statistical significance (*p* = 0.07).

In contrast, absolute dietary Mg tertiles were strongly associated with overall reported dietary intake. Median dietary Mg intake increased from 97.4 mg/day (IQR 72.0–128.6) in T1 to 583.2 mg/day (IQR 448.1–855.3) in T3, while median energy intake increased from 1127 kcal/day (IQR 927–1672) to 5385.8 kcal/day (IQR 3818–8745), respectively. Protein, carbohydrate, fat energy, fiber, omega-3, and omega-6 intakes also differed significantly across dietary Mg tertiles (all *p* < 0.001). Age decreased across increasing dietary Mg tertiles (*p* < 0.001).

Among cardiometabolic variables, significant differences across absolute dietary Mg tertiles were observed for fasting blood glucose (*p* = 0.015), HbA1c (*p* = 0.022), total cholesterol (*p* = 0.022), and HDL-C (*p* = 0.009). No significant differences were observed for BMI, WHR, systolic or diastolic blood pressure, triglycerides, vitamin D, or serum Mg (all *p* > 0.05). Although mean BMI decreased from 23.31 ± 5.6 kg/m^2^ in T1 to 21.94 ± 5.9 kg/^2^ in T3, the between-tertile difference was not statistically significant (*p* = 0.20).

### 3.5. Logistic Regression Analysis

Associations between energy-adjusted dietary Mg intake and cardiometabolic outcomes were examined using nutrient-density and residual measures (Table 6). In the primary analyses adjusted for age and sex, neither measure showed statistically significant associations with overweight/obesity, central obesity, elevated blood pressure, low HDL-C, or elevated fasting glucose. For elevated triglycerides, participants in the highest tertile of residual-adjusted Mg had nominally lower odds than those in the lowest tertile (OR: 0.40; 95% CI: 0.17–0.96; individual-comparison *p* = 0.041), consistent with the full-sample analysis in Supplementary Table S3. The corresponding association for Mg density was not statistically significant (OR: 0.66; 95% CI: 0.29–1.48; *p* = 0.31). After additional adjustment for total energy intake, the highest residual-adjusted Mg tertile remained associated with nominally lower odds of elevated triglycerides (OR: 0.38; 95% CI: 0.16–0.92). The trend tests did not remain statistically significant after FDR correction.

**Table 6:** Association of Mg Density and Mg-Adjusted Residuals with Cardiometabolic Outcomes.

	Mg-Density			*p*-Trend	FDR q	Mg Adjusted-Residual			*p*-Trend	FDR q
	T1	T2	T3			T1	T2	T3		
**Obesity (Overweight/Obese)**										
*Crude*	1	1.17 (0.62–2.24)	1.06 (0.55–2.02)	0.88	0.88	1	1.42 (0.75–2.69)	0.79 (0.41–1.55)	0.2	0.30
*Model 1*		1.04 (0.53–2.0)	0.99 (0.50–1.95)	0.75	0.75		1.24 (0.65–2.39)	0.74 (0.37–1.48)	0.38	0.46
*Model 2*		1.11 (0.57–2.17)	0.92 (0.46–1.82)	0.29	0.72		1.14 (0.59–2.22)	0.81 (0.40–1.63)	0.24	0.36
**Central Obesity**										
*Crude*	1	0.79 (0.20–3.06)	0.58 (0.14–2.53)	0.77	0.88	1	0.96 (0.27–3.46)	0.38 (0.07–2.04)	0.43	0.52
*Model 1*		1.02 (0.25–4.09)	0.80 (0.17–3.66)	0.27	0.74		1.19 (0.32–4.43)	0.50 (0.09–2.81)	0.18	0.32
*Model 2*		1.01 (0.25–1.08)	0.82 (0.17–3.79)	0.39	0.72		1.28 (0.33–4.91)	0.48 (0.08–2.72)	0.27	0.36
**Elevated Blood Pressure**										
*Crude*	1	1.75 (0.74–4.15)	1.36 (0.56–3.32)	0.43	0.88	1	1.42 (0.59–3.42)	1.62 (0.67–3.87)	0.53	0.53
*Model 1*		1.86 (0.77–4.49)	1.57 (0.62–3.96)	0.47	0.74		1.47 (0.59–3.61)	1.89 (0.76–4.67)	0.48	0.48
*Model 2*		1.88 (0.77–4.56)	1.54 (0.61–3.89)	0.60	0.72		1.42 (0.57–3.52)	1.95 (0.78–4.87)	0.59	0.59
**Low HDL-C**										
*Crude*	1	0.77 (0.41–1.45)	0.90 (0.48–1.69)	0.72	0.88	1	0.61 (0.33–1.15)	1.06 (0.56–2.01)	0.17	0.30
*Model 1*		0.79 (0.42–1.53)	0.99 (0.51–1.93)	0.62	0.74		0.63 (0.33–1.21)	1.17 (0.59–2.29)	0.21	0.32
*Model 2*		0.79 (0.41–1.53)	1.0 (0.51–1.96)	0.75	0.75		0.61 (0.32–1.19)	1.19 (0.60–2.37)	0.30	0.36
**Elevated fasting glucose**										
*Crude*	1	2.46 (0.61–9.91)	0.66 (0.11–4.05)	0.17	0.88	1	2.74 (0.69–10.7)	0.33 (0.03–3.19)	0.04	0.12
*Model 1*		2.31 (0.56–9.49)	0.64 (0.10–4.07)	0.49	0.74		2.58 (0.64–10.4)	0.31 (0.03–3.14)	0.17	0.32
*Model 2*		2.69 (0.64–11.29)	0.55 (0.08–3.53)	0.10	0.60		2.16 (0.53–8.86)	0.39 (0.04–4.07)	0.15	0.36
**Elevated triglycerides**										
*Crude*	1	1.15 (0.55–2.39)	0.79 (0.37–1.72)	0.63	0.88	1	1.49 (0.73–3.03)	0.49 (0.21–1.14)	0.02	0.12
*Model 1*		0.98 (0.46–2.10)	0.66 (0.29–1.48)	0.46	0.74		1.29 (0.62–2.69)	0.40 (0.17–0.96) *	0.03	0.18
*Model 2*		0.99 (0.47–2.11)	0.66 (0.29–1.47)	0.60	0.72		1.37 (0.65–2.88)	0.38 (0.16–0.92) *	0.04	0.24

Note: Data are presented as odds ratios (ORs) with 95% confidence intervals (CIs). T1 was used as the reference tertile. Model 1 included age and sex as covariates while Model 2 included Model 1 plus total energy. *P*-trend represents the test for trend across tertiles. * *p* < 0.05 for the individual tertile comparison vs. T1. FDR-adjusted q-values were calculated using the Benjamini–Hochberg procedure and q-values < 0.05 were considered statistically significant.

Sensitivity analyses according to energy-reporting status are presented in Supplementary Table S3. The inverse association between residual-adjusted Mg and elevated triglycerides was not statistically significant after excluding under-reporters (OR: 0.75; 95% CI: 0.23–2.44; *p* = 0.63) or over-reporters (OR: 0.39; 95% CI: 0.11–1.35; *p* = 0.14). Restriction to plausible reporters yielded only 58 participants and eight events, with non-finite confidence intervals indicating unstable estimation. Several models involving absolute dietary Mg also yielded unstable estimates. These subgroup analyses therefore provided limited evidence regarding the robustness of the full-sample association.

The event counts under the primary and IDF definitions are compared in Supplementary Table S4, and the IDF-based regression analyses are presented in Supplementary Table S5. Under the IDF criteria, the highest residual-adjusted Mg tertile had lower odds of elevated triglycerides than the lowest tertile in the age- and sex-adjusted model (OR: 0.36; 95% CI: 0.11–0.83), with a nominally significant trend across tertiles (*p*-trend = 0.018). A nominally significant trend was also observed for low HDL-C (*p*-trend = 0.008), although the tertile-specific estimates did not indicate a consistent monotonic association. No adjusted trend tests were statistically significant for Mg density. Because the IDF sensitivity analyses were not corrected for multiple comparisons, these findings should be considered exploratory.

## 4. Discussion

This cross-sectional study compared serum and dietary Mg in relation to cardiometabolic outcomes among Saudi adolescents. Three main findings emerged. First, serum Mg showed little association with the assessed cardiometabolic outcomes and was only weakly correlated with dietary Mg intake. Second, absolute dietary Mg intake was strongly correlated with total energy and other dietary intakes, and its apparent inverse association with overweight/obesity was attenuated after energy adjustment. Third, residual-adjusted dietary Mg showed a nominal inverse association with elevated triglycerides; however, the corresponding trend did not remain statistically significant after FDR correction, and analyses restricted by energy-reporting status provided limited evidence of robustness. Together, these findings highlight the distinct information provided by serum and dietary Mg measures and the importance of accounting for total energy intake when evaluating dietary Mg–cardiometabolic associations.

The prevalence of hypomagnesemia in our cohort was 37.2%. Previous studies have also reported a substantial prevalence of low Mg status in pediatric populations, although direct comparisons are limited by differences in age, adiposity, clinical characteristics, and the criteria used to define hypomagnesemia [19]. The significantly higher hypomagnesemia prevalence in boys (50%) compared with girls (31.9%) in our study shows sexual differences, which have also been observed in adult cohorts [16,29]. Possible biological and dietary differences between boys and girls may contribute to the observed sex difference, although the underlying mechanisms remain unclear [5]. Inadequate dietary Mg intake was also observed in more than 50% of the cohort and was more prevalent in boys than girls (71% vs. 54%).

The weak correlation between serum Mg and reported dietary Mg intake indicates limited concordance between these measures in this sample. This may reflect physiological regulation of circulating Mg, measurement uncertainty, and dietary reporting error; it does not by itself establish the validity of either measure as an indicator of total body Mg status [5]. In the present study, this limited concordance was reflected not only by the weak correlation between the two measures but also by the difference between the prevalence of hypomagnesemia (37.2%) and inadequate dietary Mg intake (58.5%). These findings suggest that serum Mg alone may not fully reflect Mg nutritional status in adolescents [5,6,14]. Given the small magnitude of these correlations and the absence of broader associations between serum Mg and dietary variables, these findings should be interpreted cautiously and may reflect characteristics of overall dietary intake rather than a specific relationship with these fatty acids.

In the present analysis, serum Mg tertiles were not significantly associated with the examined cardiometabolic outcomes. This finding is not uniform across the literature. In adult populations, lower serum Mg has been linked to insulin resistance, features of MetS, and cardiovascular outcomes in several studies [15,29,30]. Evidence from paediatric and adolescent cohorts, however, has been heterogeneous, with reported associations varying across populations and according to the biological measure of Mg used [19,31,32]. For example, Li et al. [30] reported inverse associations between serum Mg and insulin resistance in Chinese adults, although these associations were markedly weakened after dietary intake and inflammatory markers were considered. Wang et al. [31] reported associations between whole-blood Mg and obesity in Chinese youth, while Zhang et al. [32] found associations with MetS in rural Chinese children, highlighting the heterogeneity of findings across paediatric populations and the potential influence of differences in population characteristics, Mg assessment methods, and dietary context. The absence of significant associations in our Saudi adolescent cohort may reflect differences in population characteristics and Mg assessment compared with previous studies; however, the cross-sectional design and relatively modest sample size may also have limited the ability to detect modest associations.

The apparent inverse relationship between dietary Mg and adiposity was substantially attenuated after accounting for total energy intake. Absolute dietary Mg was modestly inversely correlated with BMI, but this association was no longer significant when Mg was expressed as nutrient density or as an energy-adjusted residual. Similarly, neither energy-adjusted measure was significantly associated with overweight/obesity in logistic regression. These findings suggest that the association observed with absolute Mg intake was largely influenced by overall food and energy intake rather than representing an independent association with Mg. This finding is broadly in contrast with evidence linking higher dietary Mg intake to more favorable metabolic outcomes in adult populations. Yang et al. [17] reported that high dietary Mg intake was associated with a 28% lower prevalence of MetS in a large Chinese cohort, with reduced insulin resistance as a partial mediator. Further support comes from an umbrella review by Veronese et al. [14], based on 43 meta-analyses, which reported inverse associations between higher dietary Mg intake and type 2 diabetes, cardiovascular disease, and all-cause mortality. Within the present sample, higher dietary Mg intake was also modestly associated with lower BMI (r = −0.158). Although based on a different measure of Mg exposure, this finding is directionally consistent with observations by Shamnani et al. [33] who reported lower serum Mg concentrations among obese individuals, and with findings by Song et al. [34], who linked greater dietary Mg intake to lower predicted atherosclerotic risk in young adults. Several mechanisms have been proposed to explain the association between dietary Mg intake and adiposity [5]. However, because dietary Mg was strongly correlated with several other dietary components in the present study, these mechanisms should not be interpreted as evidence of an independent Mg effect.

Residual-adjusted dietary Mg showed a nominal inverse association with elevated triglycerides. Participants in the highest vs. lowest tertile had lower odds in the age- and sex-adjusted model (OR: 0.40; 95% CI: 0.17–0.96; individual-comparison *p* = 0.041), whereas Mg density was not significantly associated with this outcome. The corresponding trend did not remain statistically significant after FDR correction. The inverse association was also not statistically significant after excluding under-reporters or over-reporters, and estimates among plausible reporters were unstable. The IDF-based sensitivity analysis suggested an inverse association in the same direction, but these results were exploratory because no multiplicity correction was applied. Although Mg participates in pathways relevant to lipid metabolism, including lipoprotein lipase activity, insulin signaling, and inflammatory regulation [12], these findings do not establish an independent or causal triglyceride-lowering effect of Mg.

Significant differences in fasting blood glucose and HbA1c were observed across dietary Mg tertiles in the tertile analysis, despite the absence of a significant association between dietary Mg tertiles and elevated glucose in the logistic regression analysis. Several biological mechanisms may provide a basis for an association between Mg and glucose homeostasis: Mg is required for the autophosphorylation of the insulin receptor β-subunit tyrosine kinase, a critical step in insulin signal transduction; intracellular Mg depletion impairs GLUT-4 translocation to the cell membrane, reducing insulin-stimulated glucose uptake; and Mg deficiency increases intracellular calcium, which antagonises insulin signalling and promotes insulin resistance [7,8]. Guerrero-Romero and Rodriguez-Moran [8] demonstrated in a randomized controlled trial that Mg supplementation improved β-cell function and insulin sensitivity in adults with hypomagnesemia and insulin resistance, providing biological support for a potential link between Mg and glucose homeostasis, although the population and intervention setting differ from those of the present observational study.

The strong correlations observed between dietary Mg intake and several dietary variables suggest that dietary Mg may partly reflect overall dietary quality. Consequently, the observed cardiometabolic associations may not be attributable to Mg alone but rather to the combined influence of multiple components of a broader dietary pattern. Foods that contribute to dietary Mg also supply fiber, unsaturated fats, potassium, folate, and phytochemicals, making it difficult to distinguish the specific contribution of Mg from that of co-occurring nutrients and foods. Although biological mechanisms support a potential role of Mg in cardiometabolic health [14,17], the present findings cannot distinguish the contribution of Mg itself from that of the broader dietary pattern with which Mg intake is strongly correlated. Given the strong correlations between dietary Mg and other dietary variables in the present study, however, an independent effect of Mg cannot be established. Future prospective studies, intervention trials, and dietary pattern or mediation analyses are warranted to clarify these relationships.

Data on Mg and cardiometabolic risk specifically from Saudi Arabia and the broader Middle East region are limited [35]. Naseeb [18] reported an inverse association between dietary Mg intake and BMI percentile among middle-school students participating in the HEALTHY Study, although dietary Mg intake was not significantly associated with plasma glucose concentrations. Alkazemi et al. [36] reported that women with MetS in Kuwait had markedly low dietary Mg and fiber intakes, and that Mg intake was inversely associated with waist circumference and triglycerides. Gohari-Kahou et al. [29] in Iran found that both serum and dietary Mg were inversely associated with cardiovascular outcomes in adults with MetS, though the dietary associations were generally stronger. These findings, together with the available evidence from Middle Eastern populations, suggest associations between Mg intake or status and cardiometabolic risk across populations with differing demographic and metabolic characteristics.

Strengths of this study include the simultaneous assessment of serum and dietary Mg within the same adolescent cohort, evaluation of dietary Mg using both nutrient-density and residual energy-adjustment approaches, and sensitivity analyses using IDF pediatric/adolescent cardiometabolic definitions. The comprehensive biochemical assessment and use of a culturally adapted validated FFQ further strengthened the analysis.

Several limitations warrant consideration. The cross-sectional design precludes causal inference; the observed associations between dietary Mg and cardiometabolic outcomes may reflect reverse causation (e.g., obese adolescents consuming fewer Mg-rich foods) or residual confounding by unmeasured dietary and lifestyle variables. The FFQ, while validated for the Saudi population, is subject to recall bias and may wrongly estimate absolute nutrient intakes. Serum Mg is an imperfect marker of body Mg status, and intracellular Mg measurements (erythrocyte or mononuclear cell Mg) could provide complementary information on Mg status that is not captured by serum concentrations alone. Serum Ca was not measured in the present study; consequently, the potential associations of calcium status and the calcium-to-magnesium (Ca:Mg) ratio with cardiometabolic outcomes could not be evaluated. The strong correlations between dietary Mg intake and multiple dietary variables also limit the ability to distinguish an independent association of Mg from that of the broader dietary pattern. Lastly, the high reported energy intake observed in some participants raises the possibility of dietary over-reporting or portion-size estimation error. Although dietary Mg was evaluated using nutrient-density and residual energy-adjustment methods, the residual influence of dietary reporting error, particularly on associations based on absolute Mg intake, as well as residual confounding by other dietary and lifestyle factors, including physical activity, socioeconomic status, pubertal stage, overall dietary quality, supplement use, family history, and adiposity, cannot be excluded. Lastly, 75.2% of participants were classified as implausible energy reporters using the predefined EI:BMR thresholds. Restriction to plausible reporters therefore substantially reduced the analytical sample (n = 58) and the number of elevated triglyceride events (n = 8), resulting in highly unstable estimates with very wide confidence intervals. These sensitivity analyses should consequently be considered exploratory. Lastly, four serum Mg measurements below the assay’s LoQ, including three below the LoD, were retained at their recorded values, introducing uncertainty into the serum Mg analyses. Future longitudinal designs to examine whether dietary Mg intake prospectively predicts cardiometabolic outcomes in Saudi adolescents incorporate simultaneous assessment of serum Ca and Mg and their ratio, and use randomized controlled trials of dietary interventions incorporating Mg-rich food patterns to determine whether increasing dietary Mg within a broader healthy diet improves cardiometabolic outcomes.

## 5. Conclusions

Circulating and dietary Mg provide different perspectives on Mg exposure in Saudi adolescents. Despite the high prevalence of hypomagnesemia, serum Mg showed limited associations with the cardiometabolic outcomes examined. In contrast, associations observed with absolute dietary Mg were strongly influenced by total energy and overall dietary intake, as demonstrated by their attenuation after energy adjustment. A modest inverse association was observed between residual-adjusted dietary Mg and elevated triglycerides, but the corresponding trend did not remain significant after FDR correction. This finding should therefore be regarded as exploratory and requires confirmation in larger studies with more robust dietary assessment. These findings emphasize the importance of appropriate energy adjustment when evaluating micronutrient–health relationships and support further longitudinal studies to clarify the potential role of dietary Mg in adolescent lipid metabolism and cardiometabolic health.

## Figures and Tables

**Table 1:** Clinical and Dietary Characteristics According to Serum Mg Status.

Parameters	All		*p*-Value	Girls		*p*-Value	Boys		*p*-Value
	Hypo Mg	Normal Mg		Hypo Mg	Normal Mg		Hypo Mg	Normal Mg	
N	87	147		53	113		34	34	
**Anthropometric** **Parameters**									
Age (years)	14.90 ± 1.9	14.80 ± 1.6	0.33	14.62 ± 1.9	14.88 ± 1.7	0.38	15.40 ± 1.8	14.56 ± 1.5	0.06
BMI (kg/m^2^)	23.43 ± 6.2	22.50 ± 4.7	0.21	23.11 ± 6.2	22.32 ± 4.7	0.36	24.00 ± 6.4	23.15 ± 4.7	0.55
WHR	0.83 ± 0.1	0.82 ± 0.1	0.26	0.80 ± 0.1	0.79 ± 0.1	0.91	0.90 ± 0.1	0.89 ± 0.1	0.82
Systolic BP (mmHg)	115.55 ± 14.8	115.46 ± 15.3	0.96	115.31 ± 17.5	114.77 ± 15.6	0.85	115.97 ± 8.8	117.76 ± 14.4	0.56
Diastolic BP (mmHg)	70.80 ± 10.0	71.24 ± 12.0	0.78	73.06 ± 10.1	71.56 ± 11.8	0.43	66.90 ± 8.9	70.18 ± 12.8	0.25
**Biochemical Parameters**									
FBG (mmol/L)	5.45 ± 1.8	5.26 ± 1.6	0.41	5.24 ± 1.0	5.29 ± 1.7	0.84	5.83 ± 2.7	5.17 ± 0.5	0.17
Hba1c (%)	5.11 ± 1.1	5.11 ± 0.7	0.98	5.01 ± 0.7	5.09 ± 0.8	0.55	5.29 ± 1.5	5.19 ± 0.4	0.73
TC (mmol/L)	4.75 ± 1.3	4.76 ± 1.1	0.97	4.72 ± 1.1	4.77 ± 1.2	0.81	4.82 ± 1.6	4.74 ± 1.0	0.81
HDL-C (mmol/L)	1.01 ± 0.3	1.12 ± 0.4	0.04	1.00 ± 0.3	1.09 ± 0.4	0.13	1.05 ± 0.3	1.22 ± 0.4	0.04
TG (mmol/L)	1.26 ± 0.9	1.11 ± 0.4	0.08	1.13 ± 0.5	1.11 ± 0.4	0.75	1.48 ± 1.2	1.11 ± 0.5	0.12
Vitamin D (nmol/L)	31.09 ± 14.3	31.42 ± 13.2	0.86	28.42 ± 14.0	30.25 ± 13.2	0.42	36.06 ± 13.7	35.84 ± 12.4	1.00
Serum Mg (mmol/L)	0.42 ± 0.2	1.04 ± 0.2	<0.001	0.42 ± 0.2	1.04 ± 0.2	<0.001	0.40 ± 0.2	1.03 ± 0.2	<0.001
**Dietary Parameters**									
Dietary Mg (mg/day)	189.5 (104–410)	258.4 (158–456)	0.08	228.9 (139.5–519)	254.9 (134.9–455.9)	0.92	159.7 (95.9–280.7)	263.5 (201.7–436.9)	<0.001
Energy (kcal/day)	1930 (1122–3665)	2528 (1573–4639)	0.02	2542 (1422–4107)	2661.0 (1565–4639)	0.47	1702 (1012–2974)	2338.4 (1714–4032)	0.007
Fat Energy (kcal/day)	649 (359–1336)	885 (535–1819)	0.007	808.2 (462–1535)	942.3 (545–1857)	0.35	513.5 (306–671)	756.0 (519.6–1560.5)	0.004
Protein (g/day)	74.1 (44.1–140.2)	94.4 (52.6–160.5)	0.05	76.6 (47.6–147.4)	101.3 (49.0–160.5)	0.69	64.0 (31.9–88.2)	88.7 (62.4–147.0)	0.01
Carbs (g/day)	274.5 (152–510)	327.9 (212.0–609.6)	0.08	302 (175.5–543.1)	329.7 (210.8–609.6)	0.62	246.1 (142–421.5)	323.9 (214.7–559.1)	0.04
Fiber (g/day)	19.9 (8.5–32.3)	20.8 (11.8–41.1)	0.17	20.5 (10.2–43.2)	20.5 (11.8–39.2)	0.80	17.8 (8.0–27.6)	24.2 (16.5–42.7)	0.06
Omega 3 (g/day)	0.7 (0.4–2.1)	1.3 (0.7–2.8)	0.007	1.0 (0.5–2.7)	1.4 (0.6–2.8)	0.39	0.6 (0.3–0.9)	1.0 (0.7–2.7)	0.002
Omega 6 (g/day)	4.9 (2.5–14.3)	9.4 (4.5–21.9)	0.003	6.9 (2.6–17.0)	11.0 (4.8–21.9)	0.24	3.8 (2.4–6.6)	7.3 (4.5–16.2)	0.005

Note: Data presented as mean ± SD or median (interquartile range). Statistical significance set at *p* < 0.05.

**Table 2:** Prevalence of Hypomagnesemia and Inadequate Dietary Mg Intake According to Sex and Weight Status.

Variable	Total	Girls	Boys	*p*-Value	Normal Weight	Overweight/Obese	*p*-Value
**Serum Mg Status**							
Normomagnesemia (≥0.75 mmol/L)	147 (62.8)	113 (68.1)	34 (50)	0.011	91 (63.6)	56 (61.5)	0.78
Hypomagnesemia (<0.75 mmol/L)	87 (37.2)	53 (31.9)	34 (50)		52 (36.4)	35 (38.5)	
**Dietary Mg Intake**							
Inadequate intake	137 (58.5)	89 (53.6)	48 (70.6)	0.012	76 (53.1)	61 (67.0)	0.024
Adequate intake	97 (41.5)	77 (46.4)	20 (29.4)		67 (46.9)	30 (33.0)	

Note: Data presented as n (%). Inadequate intake (≤200 mg/day for boys and girls aged 11–13; ≤340 mg/day for boys aged 14–18 and <300 mg/day for girls aged 14–18) [24].

**Table 3:** Spearman Correlation Analysis of Serum and Dietary Mg with Selected Cardiometabolic and Dietary Parameters (Overall Sample).

Parameters	Serum Mg (mmol/L)	Dietary Mg (mg/Day)	Mg Density	Mg-Adjusted Residual
Serum Mg (mmol/L)	1			
Dietary Mg (mg/day)	0.07	1		
Mg Density	−0.07	0.33 **	1	
Mg-Adjusted residual	−0.07	0.61 **	0.87 **	1
Age	−0.01	−0.23 **	0.16 *	−0.21 **
BMI	0.01	−0.16 *	−0.06	−0.11
WHR	−0.11	−0.13	0.03	0.01
Systolic BP	−0.02	−0.03	0.06	0.01
Diastolic BP	−0.04	0.01	−0.04	−0.03
FBG (mmol/L)	−0.11	0.07	0.10	0.10
HbA1c (%)	0.01	−0.03	−0.04	−0.02
TC (mmol/L)	0.05	−0.06	−0.06	−0.07
HDL-C (mmol/L)	0.11	−0.05	−0.003	−0.01
TG (mmol/L)	−0.06	−0.04	−0.004	−0.03
Vitamin D (nmol/L)	−0.03	0.02	−0.02	−0.03
Energy (kcal/day)	0.12	0.87 **	−0.13 *	0.21 **
Fat Energy (kcal/day)	0.12	0.81 **	−0.17 *	0.17 **
Protein (g/day)	0.08	0.90 **	0.08	0.40 **
Carbs (g/day)	0.11	0.84 **	−0.13 *	0.20 **
Fiber (g/day)	0.08	0.84 **	0.05	0.34 **
Omega 3 (g/day)	0.14 *	0.80 **	−0.02	0.27 **
Omega 6 (g/day)	0.16 *	0.74 **	−0.10	0.22 **

Note: Spearman correlation coefficients (r). * *p* < 0.05; ** *p* < 0.01. BMI, body mass index; WHR, waist-to-hip ratio; FBG, fasting blood glucose; TC, total cholesterol; HDL-C, HDL-cholesterol; TG, triglycerides.

**Table 4:** Spearman Correlation Analysis of Serum and Dietary Mg with Selected Cardiometabolic and Dietary Parameters, Stratified by Sex and Serum Mg Status.

Parameters	All		Girls						Boys					
			All Girls		Normomagnesemia		Hypomagnesemia		All Boys		Normomagnesemia		Hypomagnesemia	
	Serum	Dietary	Serum	Dietary	Serum	Dietary	Serum	Dietary	Serum	Dietary	Serum	Dietary	Serum	Dietary
Serum Mg (mmol/L)	1	0.07	1		1		1		1		1		1	
Dietary Mg (mg/day)	0.07	1	−0.03	1	−0.18	1	−0.01	1	0.30 *	1	0.01	1	−0.06	1
Age (years)	−0.01	−0.23 **	0.09	−0.23 **	0.15	−0.42 **	0.05	−0.15	−0.24	−0.21	0.16	−0.19	−0.13	−0.04
BMI (kg/m^2^)	0.01	−0.16 *	0.01	−0.17 *	−0.10	−0.06	0.05	−0.222 *	0.09	−0.08	0.03	−0.02	0.21	−0.1
WHR	−0.11	−0.13	−0.06	−0.12	0.05	−0.06	−0.07	−0.14	0.1	−0.04	0.07	0.14	0.09	−0.31
Systolic BP (mmHg)	−0.02	−0.03	−0.06	−0.06	−0.15	−0.16	−0.14	−0.02	0.1	0.05	−0.21	0.22	0.40 *	−0.04
Diastolic BP (mmHg)	−0.04	0.01	−0.11	0.02	0.01	−0.11	−0.12	0.04	0	−0.06	−0.29	0.08	−0.12	−0.18
FBG (mmol/L)	−0.11	0.07	−0.1	0.08	−0.04	0.11	−0.08	0.06	−0.13	0.04	−0.38 *	0.05	0.27	0.24
HbA1c (%)	0.01	−0.03	0.03	0.05	0.06	0.25	−0.06	−0.04	0.04	−0.16	−0.1	−0.24	0	−0.13
TC (mmol/L)	0.05	−0.06	0.05	−0.12	0.27	0.06	−0.01	−0.207 *	−0.01	0.09	−0.15	0.1	−0.05	0.05
HDL-C (mmol/L)	0.11	−0.05	0.09	−0.12	0.26	0.01	−0.04	−0.17	0.23	0.21	−0.32	0.29	0.14	−0.13
TG (mmol/L)	−0.06	−0.04	0	−0.01	−0.07	0.22	0.02	−0.13	−0.18	−0.08	0.03	0.07	−0.12	0.02
Vitamin D (nmol/L)	−0.03	0.02	0.02	0	−0.18	−0.02	−0.07	0.02	0.03	0.16	0.12	0.16	−0.01	0.16
Energy (kcal/day)	0.12	0.87 **	0.04	0.87 **	0	0.86 **	0	0.87 **	0.25 *	0.84 **	−0.03	0.83 **	−0.12	0.76 **
Fat Energy (kcal/day)	0.12	0.80 **	0.03	0.80 **	0	0.80 **	−0.06	0.80 **	0.22	0.76 **	−0.06	0.78 **	−0.32	0.64 **
Protein (g/day)	0.08	0.90 **	0	0.89 **	−0.11	0.91 **	−0.03	0.88 **	0.24 *	0.90 **	−0.05	0.86 **	−0.11	0.89 **
Carbs (g/day)	0.11	0.84 **	0.06	0.85 **	−0.03	0.84 **	0.06	0.85 **	0.20	0.78 **	0	0.79 **	−0.07	0.69 **
Fiber (g/day)	0.08	0.84 **	0.04	0.84 **	−0.15	0.84 **	0.08	0.85 **	0.17	0.81 **	−0.08	0.87 **	−0.07	0.70 **
Omega 3 (g/day)	0.14 *	0.78 **	0.02	0.76 **	0	0.67 **	−0.07	0.80 **	0.33 **	0.77 **	0.02	0.82 **	−0.03	0.65 **
Omega 6 (g/day)	0.16 *	0.74 **	0.06	0.74 **	0.01	0.63 **	−0.05	0.78 **	0.28 *	0.70 **	−0.03	0.75 **	−0.1	0.57 **

Note: Data are presented as Spearman correlation coefficients (r). * *p* < 0.05, ** *p* < 0.01.

**Table 5:** Clinical and Dietary Characteristics Across Serum and Dietary Mg Tertiles.

Parameters	Serum Mg Tertiles			*p*-Value	Dietary Mg Tertiles			*p*-Value
	T1	T2	T3		T1	T2	T3	
n (M/F)	77 (31/46)	75 (19/56)	82 (18/64)		77 (21/56)	79 (31/48)	78 (16/62)	
Age (years)	14.71 ± 1.8	15.01 ± 1.8	14.79 ± 1.5	0.54	15.35 ± 1.8	14.87 ± 1.6	14.32 ± 1.6	<0.001
BMI (kg/m^2^)	23.50 ± 6.3	22.36 ± 5.3	22.67 ± 4.2	0.41	23.31 ± 5.6	23.26 ± 4.2	21.94 ± 5.9	0.2
WHR	0.83 ± 0.1	0.82 ± 0.1	0.81 ± 0.1	0.31	0.84 ± 0.1	0.83 ± 0.1	0.80 ± 0.1	0.06
Systolic BP (mmHg)	115.86 ± 14.7	115.08 ± 13.1	115.55 ± 17.3	0.95	114.72 ± 15.1	117.0 ± 14.1	114.77 ± 16.2	0.57
Diastolic BP (mmHg)	70.78 ± 10.0	71.11 ± 9.5	71.33 ± 13.8	0.96	70.58 ± 10.8	72.55 ± 10.6	70.13 ± 12.5	0.37
FBG (mmol/L)	5.53 ± 1.9	5.05 ± 0.6	5.41 ± 2.0	0.18	5.09 ± 0.7	5.79 ± 2.7	5.14 ± 0.5	0.015
HbA1c (%)	5.15 ± 1.1	5.04 ± 0.4	5.15 ± 0.9	0.7	5.01 ± 0.5	5.35 ± 1.3	5.0 ± 0.6	0.022
TC (mmol/L)	4.76 ± 1.3	4.77 ± 1.1	4.75 ± 1.2	0.99	4.82 ± 1.4	5.0 ± 1.4	4.47 ± 0.7	0.022
HDL-C (mmol/L)	1.01 ± 0.3	1.12 ± 0.4	1.12 ± 0.4	0.12	1.10 ± 0.4	1.17 ± 0.4	0.99 ± 0.2	0.009
TG (mmol/L)	1.28 ± 0.9	1.12 ± 0.5	1.10 ± 0.4	0.17	1.16 ± 0.6	1.26 ± 0.8	1.07 ± 0.4	0.18
Vitamin D (nmol/L)	31.42 ± 14.8	31.99 ± 14.7	30.57 ± 11.4	0.81	30.52 ± 14.0	32.16 ± 13.3	31.26 ± 13.6	0.77
Serum Mg (mmol/L)	0.38 ± 0.2	0.84 ± 0.1	1.18 ± 0.2	<0.001	0.75 ± 0.4	0.86 ± 0.4	0.80 ± 0.3	0.17
Dietary Mg (mg/day)	203.9 (108.6–416)	233.8 (120–456)	280 (174–433)	0.31	97.4 (72.0–128.6)	242.8 (200.1–293.9)	583.2 (448.1–855.3)	<0.001
Energy (kcal/day)	1930 (1122–3649)	2308 (1382–4922)	2731 (1675–4294)	0.07	1127 (927–1672)	2312.5 (1714–2998)	5385.8 (3818–8745)	<0.001
Fat Energy (kcal/day)	603.3 (362–1212)	840.3 (517–1989)	904.9 (582.4–1609)	0.03	440.2 (291–593)	752.1 (539–1061)	1963.3 (1336–3057)	<0.001
Protein (g/day)	74.3 (44.1–140)	80.1 (46.9–151)	100.0 (57.3–147)	0.21	42.2 (24.7–52.6)	84.3 (63.5–106.1)	195.8 (139.5–343.3)	<0.001
Carbs (g/day)	283.6 (152–501)	295.3 (166–559)	369.6 (215.8–616.9)	0.18	152.5 (100–234)	306.4 (225.6–435.5)	669.1 (455–1204.9)	<0.001
Fiber (g/day)	21.1 (8.6–32.8)	18.9 (10.3–34.2)	21.8 (12.3–42.9)	0.38	9.23 (4.8–14.0)	21.00 (15.0–27.8)	50.47 (31.1–81.0)	<0.001
Omega 3 (g/day)	0.7 (0.4–1.8)	1.1 (0.5–3.3)	1.4 (0.7–2.5)	0.013	0.44 (0.3–0.7)	0.94 (0.7–1.7)	3.13 (1.7–6.8)	<0.001
Omega 6 (g/day)	4.8 (2.6–12.2)	8.5 (3.1–24.1)	11.0 (5.3–16.9)	0.007	2.99 (1.5–5.7)	7.35 (4.0–11.5)	23.93 (14.3–40.9)	<0.001

Note: Data presented as mean ± SD or median (interquartile range). T1 represents the lowest tertile and T3 the highest tertile. Statistical significance was considered at *p* < 0.05.

## Data Availability

The data presented in this study are available on reasonable request from the corresponding author. The data are not publicly available due to participant privacy considerations.

## Supplementary Materials

The following supporting information can be downloaded at: https://www.mdpi.com/article/10.3390/nu18193302/s1.

## References

[1] Lobstein T., Jackson-Leach R., Moodie M.L., Hall K.D., Gortmaker S.L., Swinburn B.A., James W.P., Wang Y., McPherson K. (2015). Child and adolescent obesity: Part of a bigger picture. Lancet.

[2] Al-Hazzaa H.M., Abahussain N.A., Al-Sobayel H.I., Qahwaji D.M., Musaiger A.O. (2011). Physical activity, sedentary behaviors and dietary habits among Saudi adolescents relative to age, gender and region. Int. J. Behav. Nutr. Phys. Act..

[3] Alhussain M.H., Macdonald I.A., Taylor M.A. (2016). Irregular meal-pattern effects on energy expenditure, metabolism, and appetite regulation: A randomized controlled trial in healthy normal-weight women. Am. J. Clin. Nutr..

[4] Bao W., Srinivasan S.R., Berenson G.S. (1996). Persistent elevation of plasma insulin levels is associated with increased cardiovascular risk in children and young adults. The Bogalusa Heart Study. Circulation.

[5] de Baaij J.H., Hoenderop J.G., Bindels R.J. (2015). Magnesium in man: Implications for health and disease. Physiol. Rev..

[6] Volpe S.L. (2013). Magnesium in disease prevention and overall health. Adv. Nutr..

[7] Barbagallo M., Dominguez L.J. (2015). Magnesium and type 2 diabetes. World J. Diabetes.

[8] Guerrero-Romero F., Rodriguez-Moran M. (2011). Magnesium improves the beta-cell function to compensate variation of insulin sensitivity: Double-blind, randomized clinical trial. Eur. J. Clin. Investig..

[9] Guerrero-Romero F., Rodriguez-Moran M. (2009). The effect of lowering blood pressure by magnesium supplementation in diabetic hypertensive adults with low serum magnesium levels: A randomized, double-blind, placebo-controlled clinical trial. J. Hum. Hypertens..

[10] Shechter M. (2010). Magnesium and cardiovascular system. Magnes. Res..

[11] Marabelli C., Santiago D.J., Priori S.G. (2025). The Yin and Yang of Heartbeats: Magnesium-Calcium Antagonism Is Essential for Cardiac Excitation-Contraction Coupling. Cells.

[12] Tangvoraphonkchai K., Davenport A. (2018). Magnesium and Cardiovascular Disease. Adv. Chronic Kidney Dis..

[13] Dibaba D.T., Xun P., He K. (2014). Dietary magnesium intake is inversely associated with serum *C*-reactive protein levels: Meta-analysis and systematic review. Eur. J. Clin. Nutr..

[14] Veronese N., Demurtas J., Pesolillo G., Celotto S., Barnini T., Calusi G., Caruso M.G., Notarnicola M., Reddavide R., Stubbs B. (2020). Magnesium and health outcomes: An umbrella review of systematic reviews and meta-analyses of observational and intervention studies. Eur. J. Nutr..

[15] Kuppusamy S., Dhanasinghu R., Sakthivadivel V., Kaliappan A., Gaur A., Balan Y., Tadi L.J., Sundaramurthy R. (2022). Association of Serum Magnesium with Insulin Indices in Patients with Type 2 Diabetes Mellitus. Maedica.

[16] Ma J., Folsom A.R., Melnick S.L., Eckfeldt J.H., Sharrett A.R., Nabulsi A.A., Hutchinson R.G., Metcalf P.A. (1995). Associations of serum and dietary magnesium with cardiovascular disease, hypertension, diabetes, insulin, and carotid arterial wall thickness: The ARIC study. Atherosclerosis Risk in Communities Study. J. Clin. Epidemiol..

[17] Yang N., He L., Li Y., Xu L., Ping F., Li W., Zhang H. (2020). Reduced Insulin Resistance Partly Mediated the Association of High Dietary Magnesium Intake with Less Metabolic Syndrome in a Large Chinese Population. Diabetes Metab. Syndr. Obes..

[18] Naseeb M., Milliron B.J., Bruneau M.L., Sukumar D., Foster G.D., Smith S.A., Volpe S.L. (2021). Dietary magnesium intake in relation to body mass index and glycemic indices in middle school students from the HEALTHY Study. Nutr. Health.

[19] Nassar M., Fouad N., Taha F., Elkholy H. (2024). Insulin Sensitivity in Relation to Serum Magnesium in Obese Children. QJM Int. J. Med..

[20] Amer O.E., Sabico S., Khattak M.N.K., Alnaami A.M., Aljohani N.J., Alfawaz H., AlHameidi A., Al-Daghri N.M. (2021). Increasing Prevalence of Pediatric Metabolic Syndrome and Its Components among Arab Youth: A Time-Series Study from 2010–2019. Children.

[21] Sabico S., Al-Daghri N.M., Alenad A., Al-Saleh Y., Khattak M.N.K., Wani K., Alnaami A.M., Reginster J.Y., Alokail M.S., Cavalier E. (2025). Sexual dimorphism in LC-MS/MS-based vitamin D metabolite profiling and nutrition-acquired biochemical osteomalacia among adolescents. Front. Nutr..

[22] De la Cruz-Góngora V., Gaona B., Villalpando S., Shamah-Levy T., Robledo R. (2012). Anemia and iron, zinc, copper and magnesium deficiency in Mexican adolescents: National Health and Nutrition Survey 2006. Salud Publica Mex..

[23] Gosadi I.M., Alatar A.A., Otayf M.M., AlJahani D.M., Ghabbani H.M., AlRajban W.A., Alrsheed A.M., Al-Nasser K.A. (2017). Development of a Saudi Food Frequency Questionnaire and testing its reliability and validity. Saudi Med. J..

[24] Otten J.J., Hellwig J.P., Meyers L.D. (2006). DRI, Dietary Reference Intakes: The Essential Guide to Nutrient Requirements.

[25] Cook S., Weitzman M., Auinger P., Nguyen M., Dietz W.H. (2003). Prevalence of a metabolic syndrome phenotype in adolescents: Findings from the third National Health and Nutrition Examination Survey, 1988–1994. Arch. Pediatr. Adolesc. Med..

[26] Wani K., Khattak M.N.K., Saadawy G.M., Al-Attas O.S., Alokail M.S., Al-Daghri N.M. (2023). Sex-Specific Cut-Offs of Single Point Insulin Sensitivity Estimator (SPISE) in Predicting Metabolic Syndrome in the Arab Adolescents. Diagnostics.

[27] Kuczmarski R.J., Ogden C.L., Grummer-Strawn L.M., Flegal K.M., Guo S.S., Wei R., Mei Z., Curtin L.R., Roche A.F., Johnson C.L. (2000). CDC growth charts: United States. Adv. Data.

[28] Zimmet P., Alberti K.G., Kaufman F., Tajima N., Silink M., Arslanian S., Wong G., Bennett P., Shaw J., Caprio S. (2007). The metabolic syndrome in children and adolescents—An IDF consensus report. Pediatr. Diabetes.

[29] Gohari-Kahou M., Darroudi S., Sabery Karimian M., Parizadeh S.M., Asadi Z., Javandoost A., Safarian M., Mouhebati M., Ebrahimi M., Ferns G. (2020). The association between serum and dietary magnesium with cardiovascular disease risk factors in Iranian adults with metabolic syndrome. Transl. Metab. Syndr. Res..

[30] Li W., Jiao Y., Wang L., Wang S., Hao L., Wang Z., Wang H., Zhang B., Ding G., Jiang H. (2022). Association of Serum Magnesium with Insulin Resistance and Type 2 Diabetes among Adults in China. Nutrients.

[31] Wang T., Wang L., Ma N., Gu S., Jiang D., Li J., Guo C., Peng F., Zhao S., Li H. (2022). Whole-blood magnesium and blood lipids are individually and jointly associated with an elevated likelihood of youngsters being overweight or obese: A matched case-control study using the propensity score. Nutrition.

[32] Zhang H., Man Q., Song P., Li S., Liu X., Wang L., Li Y., Hu Y., Yang L. (2021). Association of whole blood copper, magnesium and zinc levels with metabolic syndrome components in 6-12-year-old rural Chinese children: 2010–2012 China National Nutrition and Health Survey. Nutr. Metab..

[33] Shamnani G., Rukadikar C.A., Gupta V., Singh S., Tiwari S., Bhartiy S.S., Sharma P. (2018). Serum magnesium in relation with obesity. Natl. J. Physiol. Pharm. Pharmacol..

[34] Song Q., Song L., Liu H., Tan H., Yang B., Fan C., Liu L. (2024). Association of magnesium intake with predicted atherosclerotic lesions and cardiovascular risk in young adults based on PDAY score: A cross-sectional study. BMC Public Health.

[35] Al-Daghri N.M., Yakout S.M., Hussain S.D., Alnaami A.M., Veronese N., Barbagallo M., Sabico S. (2025). Hypomagnesemia in adults with type 2 diabetes mellitus in Riyadh, Saudi Arabia: A cross-sectional study. Medicine.

[36] Alkazemi D.U.Z., Zafar T.A., Alsouri N.Y., Aljahdali A.A., Kubow S. (2024). Low dietary magnesium and fiber intakes among women with metabolic syndrome in Kuwait. Front. Nutr..

