# Supplementary material for: Serum vs. Dietary Magnesium and Cardiometabolic Outcomes in Saudi Adolescents
Source: Nutrients. 2026 Oct 8;18(19):3302. doi: 10.3390/nu18193302 (PMC13666817; doi:10.3390/nu18193302)
Supplement: Supplementary file 1 [file nutrients-18-03302-s001.zip › nutrients-4563732-supplementary.pdf]

**Supplementary Materials**

**Supplementary Methods**

**S1.** Definition of Implausible Energy Reporting

Basal metabolic rate (BMR) was estimated using the FAO/WHO/UNU weight-based equations for adolescents aged 10–18 years: BMR = 17.5 × weight (kg) + 651 kcal/day for boys and BMR = 12.2 × weight (kg) + 746 kcal/day for girls. The ratio of reported energy intake (EI) to estimated BMR was calculated as EI:BMR = reported energy intake (kcal/day) / estimated BMR (kcal/day). Participants were classified using Goldberg-type cut-offs: under-reporters were defined as EI:BMR <1.35, plausible reporters as EI:BMR 1.35–<2.40, and over-reporters as EI:BMR ≥2.40. Accordingly, implausible energy reporters were defined as participants with EI:BMR <1.35 or ≥2.40.

Among the 234 participants assessed for energy-reporting plausibility, 102 (43.6%) were classified as under-reporters, 58 (24.8%) as plausible reporters, and 74 (31.6%) as over-reporters. Thus, 176 participants (75.2%) were classified as implausible energy reporters and excluded from the relevant analyses, leaving 58 participants with plausible energy reporting. Compared across reporting categories, significant differences were observed in age (under-reporters: 15.3 ± 1.8 years; plausible reporters: 14.8 ± 0.6 years; over-reporters: 14.3 ± 1.6 years; $p<0.001$) and BMI (23.9 ± 5.7, 22.8 ± 4.1, and 21.4 ± 5.3 kg/m$^2$, respectively; $p=0.009$). From the classification method, total energy intake and dietary Mg intake also differed significantly across categories (both $p<0.001$). In contrast, serum Mg concentrations were comparable among under-, plausible, and over-reporters (0.77 ± 0.4, 0.84 ± 0.4, and 0.84 ± 0.3, respectively; $p=0.29$).

**Supplementary Tables**

**Supplementary Table S1.** Distribution of serum Mg

| Statistic | Serum Mg (mmol/L) | Serum Mg (mmol/L)# |
|---|---|---|
| N | 234 | 230 |
| Minimum | 0.01 | 0.06 |
| 5th percentile | 0.15 | 0.17 |
| 25th percentile | 0.55 | 0.57 |
| Mean | 0.81 | 0.82 |
| Std. Dev | 0.37 | 0.35 |
| Median | 0.86 | 0.865 |
| 75th percentile | 1.07 | 1.07 |
| 95th percentile | 1.37 | 1.39 |
| Maximum | 1.76 | 1.76 |
| IQR | 0.55–1.07 | 0.57–1.07 |

**Note:** Four measurements below LoQ removed

**Supplementary Table S2.** Distribution of reported total energy intake

| Parameter | Energy intake, kcal/day |
|---|---|
| N | 234 |
| Minimum | 87.7 |
| 5th percentile | 548.3 |
| 25th percentile | 1,413.4 |
| Mean | 3664.38 |
| Standard Deviation | 4034.26 |
| Median | 2,358.8 |
| 75th percentile | 4,152.9 |
| 95th percentile | 10,763.9 |
| 99th percentile | 19,808.8 |
| Maximum | 29,350.4 |

**Supplementary table S3**. Sensitivity analysis of the association between dietary Mg and hypertriglyceridemia according to energy-reporting status.

| | | N | Events | OR (95%) | *p*-value |
|---|---|---|---|---|---|
| Mg Dietary Intake | All | 234 | 53 | T1=Reference | |
| | T2 | | | 0.77 (0.37-1.62) | 0.49 |
| | T3 | | | 0.44 (0.19-0.98) | 0.046 |
| | Excluding Under R | 132 | 22 | 1 | |
| | T2 | | | $42.2*10^{7}$ (0-∞) | 0.99 |
| | T3 | | | $27.6*10^{7}$ (0-∞) | 0.99 |
| | Excluding Over R | 160 | 39 | 1 | |
| | T2 | | | 0.79 (0.36-1.71) | 0.54 |
| | T3 | | | 0.31 (0.06-1.48) | 0.14 |
| | Plausible R Only | 58 | 8 | 1 | |
| | T2 | | | $45.2*10^{7}$ (0-∞) | 0.99 |
| | T3 | | | $29.5*10^{7}$ (0-∞) | 0.99 |
| Mg Density | All | 234 | 53 | 1 | |
| | T2 | | | 0.98 (0.46-2.10) | 0.96 |
| | T3 | | | 0.66 (0.29-1.48) | 0.31 |
| | Excluding Under R | 132 | 22 | 1 | |
| | T2 | | | 1.14 (0.36-3.67) | 0.82 |
| | T3 | | | 0.88 (0.24-3.10) | 0.84 |
| | Excluding Over R | 160 | 39 | 1 | |
| | T2 | | | 1.56 (0.64-3.81) | 0.33 |
| | T3 | | | 0.79 (0.30-2.04) | 0.62 |
| | Plausible R Only | 58 | 8 | 1 | |
| | T2 | | | $54.4*10^{7}$ (0-∞) | 0.99 |
| | T3 | | | $27.2*10^{7}$ (0-∞) | 0.99 |
| Mg residuals adjusted | All | 234 | 53 | 1 | |
| | T2 | | | 1.29 (0.63-2.69) | 0.48 |
| | T3 | | | 0.40 (0.17-0.96) | 0.041 |
| | Excluding Under R | 132 | 22 | 1 | |
| | T2 | | | 1.64 (0.47-5.77) | 0.44 |
| | T3 | | | 0.75 (0.23-2.44) | 0.63 |
| | Excluding Over R | 160 | 39 | 1 | |
| | T2 | | | 1.55 (0.67-3.56) | 0.30 |
| | T3 | | | 0.39 (0.11-1.35) | 0.14 |
| | Plausible R Only | 58 | 8 | 1 | |
| | T2 | | | $51.7*10^{7}$ (0-∞) | 0.99 |
| | T3 | | | $23.7*10^{7}$ (0-∞) | 0.99 |

**Note**: Data are presented as odds ratios (ORs) with 95% confidence intervals (CIs) from logistic regression models adjusted for age and sex, with T1 as the reference category. Under-reporters were defined as EI:BMR <1.35, plausible reporters as EI:BMR 1.35–<2.40, and over-reporters as EI:BMR ≥2.40. EI:BMR denotes the ratio of reported energy intake to estimated basal metabolic rate; "R" denotes reporters. N represents complete cases in each model, and events represent participants with hypertriglyceridemia under the primary definition. Extremely large estimates or non-finite confidence intervals in some subgroup analyses indicate sparse data and unstable estimation and should not be interpreted as reliable associations.

**Supplementary Table S4**. Sensitivity analyses of associations between energy-adjusted dietary magnesium intake and cardiometabolic outcomes using IDF pediatric/adolescent definitions.

| **Metabolic component** | **N (%) IDF [28] (sensitivity analysis only)** | **N (%) Cook' Definition [25] (primary analysis)** |
|---|---|---|
| Central obesity | 23 (9.8) | 12 (5.1) |
| Elevated triglycerides | 31 (13.2) | 53 (22.6) |
| Low HDL-C | 151 (64.5) | 128 (54.7) |
| Elevated blood pressure | 39 (16.7) | 39 (16.7) |
| Elevated fasting glucose | 40 (17.1) | 12 (5.1) |
| Metabolic Syndrome | 5 (2.1) | 2 (0.9) |

**Supplementary Table S5.** Sensitivity analysis of associations between energy-adjusted dietary magnesium intake and cardiometabolic outcomes using IDF pediatric/adolescent definitions.

| | **Mg -density** | | | **P-Trend-** | **Mg adjusted-residual** | | | **P-Trend-** |
|---|---|---|---|---|---|---|---|---|
| IDF Definition | **T1** | **T2** | **T3** | | **T1** | **T2** | **T3** | |
| **Central Obesity** | | | | | | | | |
| *Crude* | 1 | 1.37 (0.45-4.16) | 1.56 (0.53-4.63) | 0.71 | 1 | 1.69 (0.58-4.90) | 1.18 (0.38-3.69) | 0.59 |
| *Adjusted* | | 1.39 (0.45-4.34) | 1.82 (0.59-5.61) | 0.61 | | 1.67 (0.56-4.97) | 1.36 (0.42-4.39) | 0.65 |
| **Elevated Blood Pressure** | | | | | | | | |
| *Crude* | 1 | 1.75 (0.74-4.15) | 1.36 (0.56-3.32) | 0.43 | 1 | 1.42 (0.59-3.42) | 1.62 (0.67-3.87) | 0.53 |
| *Adjusted* | | 1.86 (0.77-4.49) | 1.57 (0.62-3.96) | 0.47 | | 1.47 (0.59-3.61) | 1.89 (0.76-4.67) | 0.48 |
| **Low HDL-C** | | | | | | | | |
| *Crude* | 1 | 0.65 (0.33-1.29) | 0.95 (0.46-1.93) | 0.06 | 1 | 0.46 (0.24-0.90)* | 0.88 (0.44-1.76) | 0.045 |
| *Adjusted* | | 0.57 (0.29-1.11) | 0.79 (0.40-1.6) | 0.24 | | 0.51 (0.26-1.03) | 1.05 (0.51-2.17) | 0.008 |
| **Elevated fasting glucose** | | | | | | | | |
| *Crude* | 1 | **2.63 (1.07-6.46)*** | 1.91 (0.75-4.86) | 0.092 | 1 | **2.68 (1.09-6.58)*** | 1.75 (0.68-4.50) | 0.08 |
| *Adjusted* | | 2.18 (0.87-5.46) | 1.56 (0.59-4.08) | 0.08 | | 2.24 (0.89-5.55) | 1.46 (0.55-3.84) | 0.07 |
| **Elevated triglycerides** | | | | | | | | |
| *Crude* | 1 | 1.48 (0.62-3.58) | 0.67 (0.24-1.86) | 0.25 | 1 | 1.81 (0.77-4.25) | 0.37 (0.11-1.23) | **0.009** |
| *Adjusted* | | 1.30 (0.53-3.22) | 0.65 (0.23-1.87) | 0.17 | | 1.55 (0.64-3.74) | 0.36 (0.11-0.83) | **0.018** |

**Note**: Data are presented as odds ratios (ORs) with 95% confidence intervals (CIs) from logistic regression models, with T1 as the reference category. Adjusted models included age and sex. Cardiometabolic outcomes were defined using the IDF pediatric/adolescent criteria for sensitivity analyses. P-trend denotes the test for linear trend across tertiles. *p<0.05 for the individual tertile comparison versus T1. All *p*-values are nominal, without adjustment for multiple testing. Findings should therefore be interpreted as exploratory.

**Supplementary Figure**

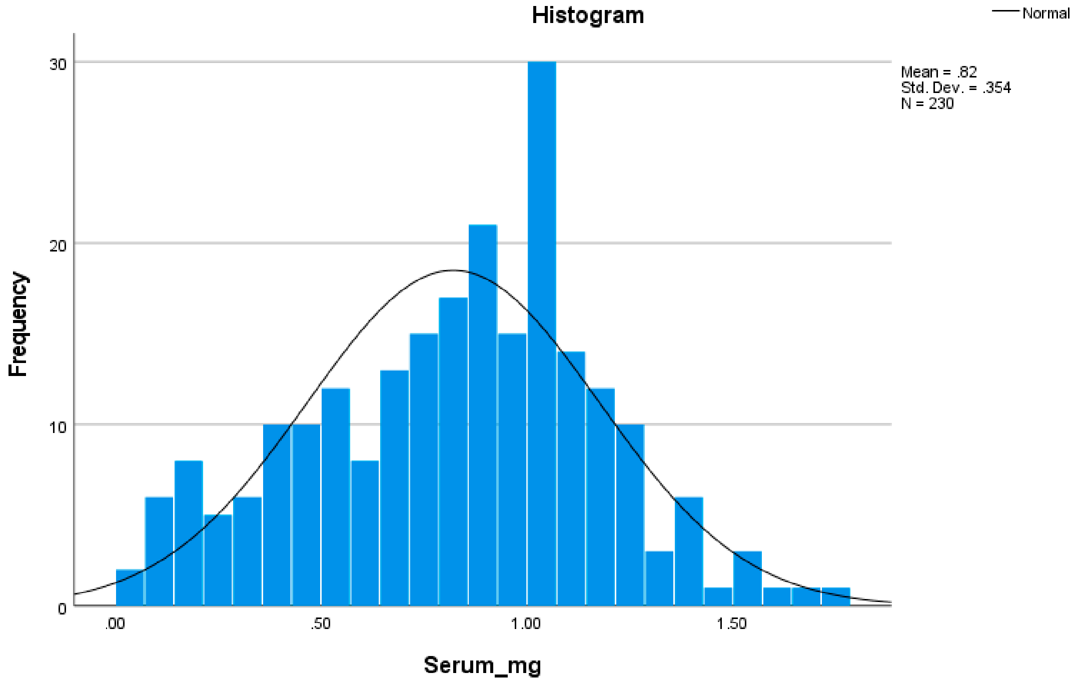


**Figure S1**. Histogram plot of serum Mg
